# Supplementary material for: Structure of a distinct β-barrel assembly machinery complex in the Bacteroidota
Source: Nat Microbiol. 2025 Oct 1;10(11):2845–59. doi: 10.1038/s41564-025-02132-2 (PMC12578637; doi:10.1038/s41564-025-02132-2)
Supplement: Supplementary file 1 — Supplementary Figs. 1–5, Supplementary Tables 2, 6 and 7, and Supplementary Discussion. [file 41564_2025_2132_MOESM1_ESM.pdf]

nature microbiology

Supplementary information https://doi.org/10.1038/s41564-025-02132-2

Structure of a distinct β-barrel assembly machinery complex in the Bacteroidota

In the format provided by the authors and unedited

Discovery of a distinct BAM complex in the Bacteroidota

Supplementary Information

Augustinas Silale[#1], Mariusz Madej[#2*], Katarzyna Mikruta[2,3], Andrew M. Frey[1], Adam J. Hart[1], Arnaud Baslé[1], Carsten Scavenius[4], Jan J. Enghild[4], Matthias Trost[1], Robert P. Hirt[1] and Bert van den Berg[1*]

[#]Authors contributed equally to this work

[1]Biosciences Institute, Faculty of Medical Sciences, Newcastle University, Newcastle upon Tyne, NE2 4HH, UK

[2]Department of Microbiology, Faculty of Biochemistry, Biophysics and Biotechnology, Jagiellonian University, 30-387 Krakow, Poland

[3]Doctoral School of Exact and Natural Sciences, Jagiellonian University, 30-348 Krakow, Poland

[4]Interdisciplinary Nanoscience Center (iNANO), Aarhus University, 8000 Aarhus C, Denmark.

* To whom correspondence should be addressed.

Email: bert.van-den-berg@newcastle.ac.uk

mariusz.madej@uj.edu.pl

Supplementary Figures

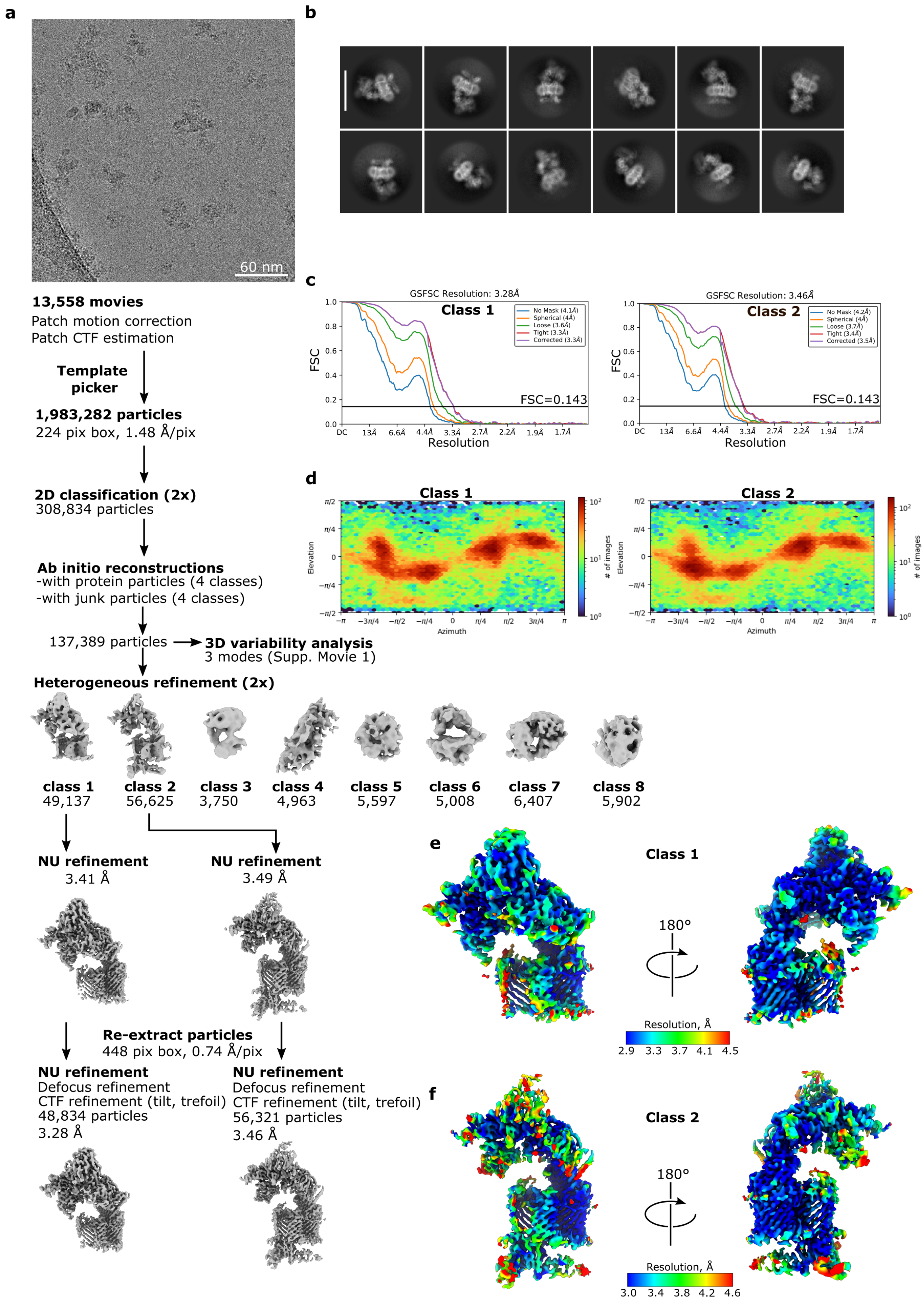

Supplementary Figure 1. BtBAM cryo-EM data processing workflow. **a**, Representative motion-corrected movie (n=13,558) and cryoSPARC data processing workflow. 3D variability analysis results are shown in Supplementary Movie 1. **b**, Representative 2D class averages. The white bar represents a length of ~165 Å. **c**, Global gold-standard Fourier shell correlation (FSC) curves. **d**, Viewing direction distribution plots. **e** and **f**, Local resolution estimation for the final class 1 and class 2 maps, respectively.

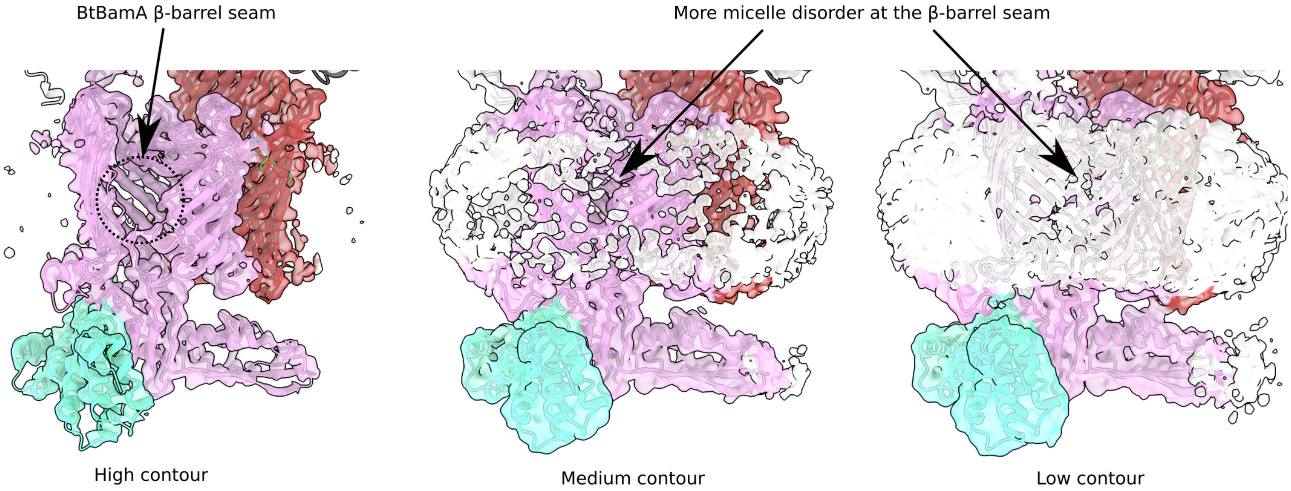

Supplementary Figure 2. Increased disorder at the BtBamA β-barrel seam. The BtBamADG model (cartoon) overlaid with the cryo-EM class 2 map (transparent surface) is displayed at three different contour levels: high, medium and low. As the threshold is lowered, the detergent micelle density does not appear uniformly around the BamA β-barrels. The density is weaker at the β-barrel seam, suggesting that there is more disorder in this region.

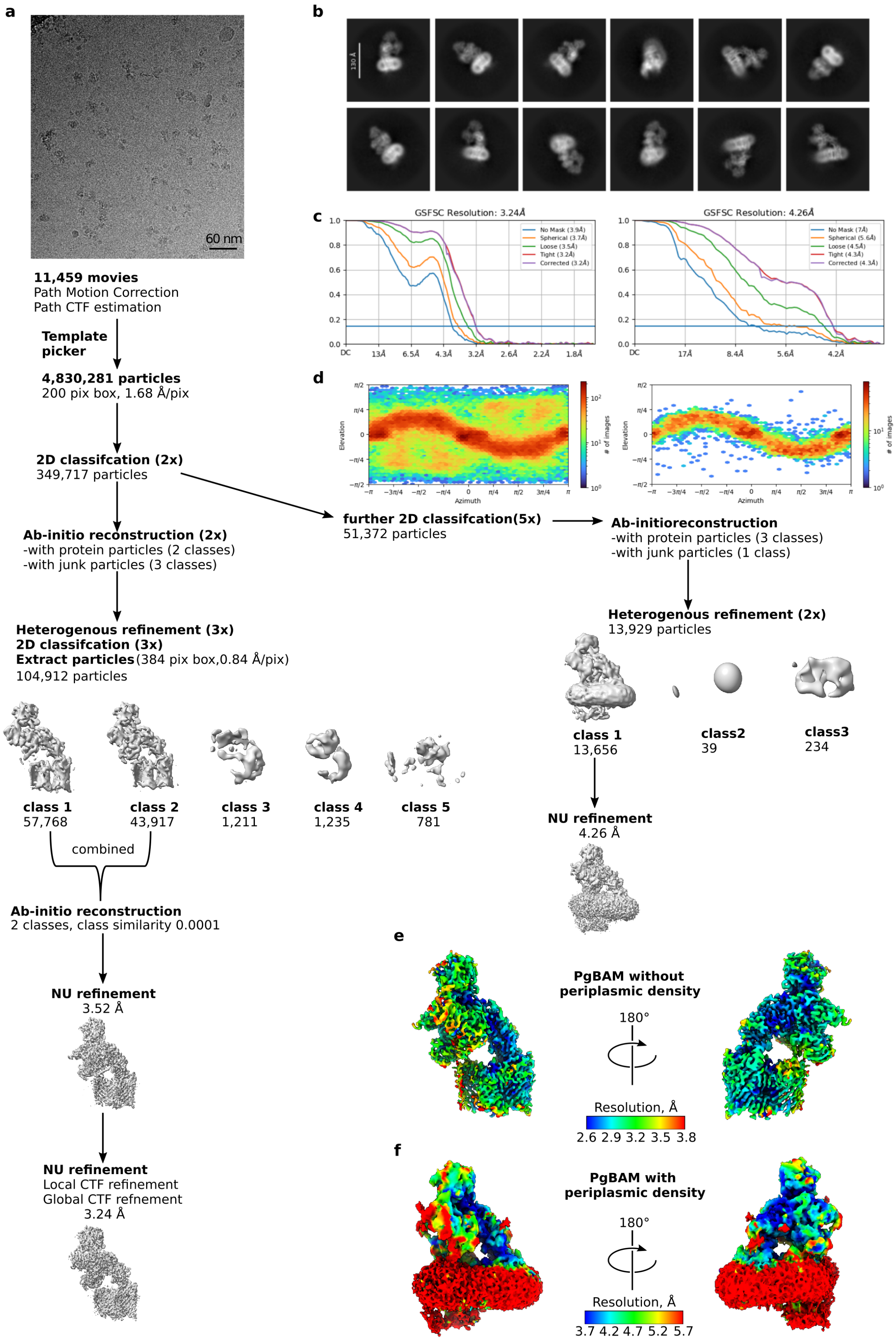
a
60 nm
11,459 movies
Path Motion Correction
Path CTF estimation
Template picker
4,830,281 particles
200 pix box, 1.68 Å/pix
2D classifcation (2x)
349,717 particles
Ab-initio reconstruction (2x)
-with protein particles (2 classes)
-with junk particles (3 classes)
Heterogenous refinement (3x)
2D classifcation (3x)
Extract particles (384 pix box,0.84 Å/pix)
104,912 particles
class 1
57,768
class 2
43,917
class 3
1,211
class 4
1,235
class 5
781
combined
Ab-initio reconstruction
2 classes, class similarity 0.0001
NU refinement
3.52 Å
NU refinement
Local CTF refinement
Global CTF refnement
3.24 Å
b
130 Å
c
GSFSC Resolution: 3.24Å
No Mask (3.9Å)
Spherical (3.7Å)
Loose (3.5Å)
Tight (3.2Å)
Corrected (3.2Å)
GSFSC Resolution: 4.26Å
No Mask (7Å)
Spherical (5.6Å)
Loose (4.5Å)
Tight (4.3Å)
Corrected (4.3Å)
d
Elevation
Azimuth
# of images
further 2D classifcation(5x)
51,372 particles
Ab-initioreconstruction
-with protein particles (3 classes)
-with junk particles (1 class)
Heterogenous refinement (2x)
13,929 particles
class 1
13,656
class2
39
class3
234
NU refinement
4.26 Å
e
PgBAM without periplasmic density
180°
Resolution, Å
2.6 2.9 3.2 3.5 3.8
f
PgBAM with periplasmic density
180°
Resolution, Å
3.7 4.2 4.7 5.2 5.7

45

Supplementary Figure 3. PgBAM cryo-EM data processing workflow. **a**, Representative motion-corrected movie (n=11,459) and cryoSPARC data processing workflow. **b**, Representative 2D class averages. The white bar represents a length of 130 Å. **c**, Global gold-standard Fourier shell correlation (FSC) curves. **d**, Viewing direction distribution plots. **e** and **f**, Local resolution estimation for the final reconstructions without and with periplasmic density, respectively.

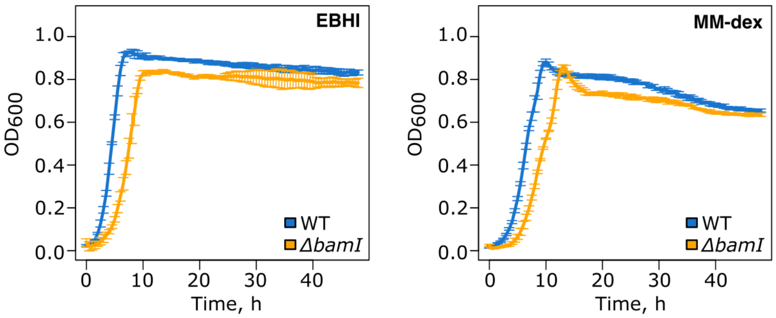

Supplementary Figure 4. Growth curves of *B. theta* Δ*bamI* strain. Cells were cultured either in EBHI or in minimal medium supplemented with 0.4% dextran 40 and 10% BHI (MM-dex). Each trace is an average of n=3 technical repeats. The error bars show the standard deviation. Growth curves are representative for 2 biological replicates.

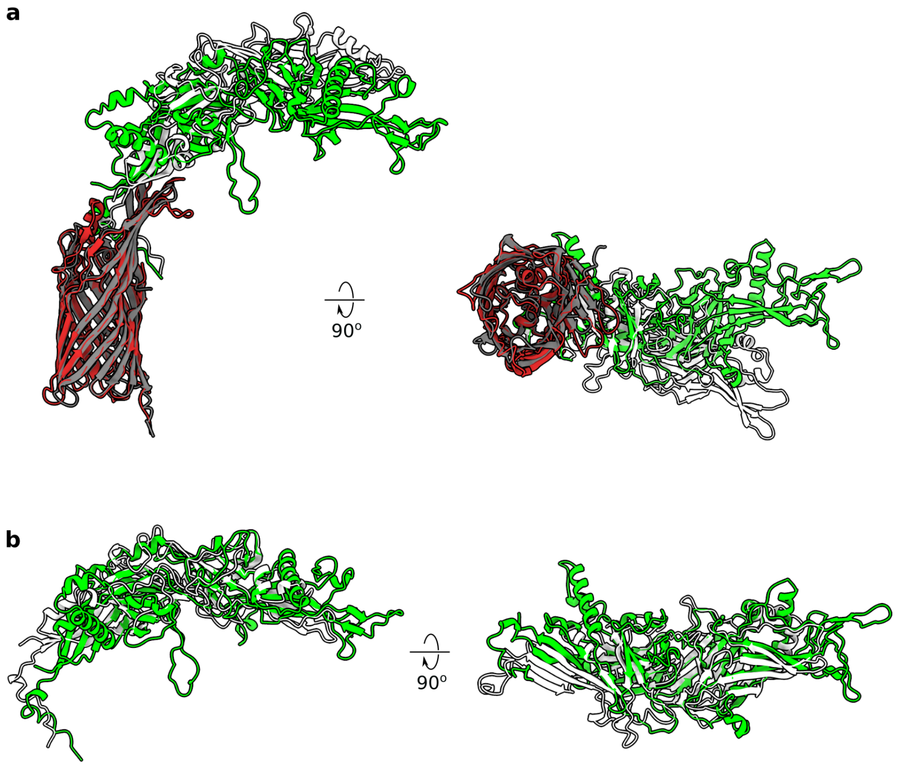

Supplementary Figure 5. Structural comparison of BtBamGH and BtBamG2H2 (Bt1785-86). **a**, Superposition of the BtBamGH cryo-EM structure (red, green) and AF3[1] prediction of the Bt1785-86 complex (grey, white), renamed BamG2H2. The superposition was generated using Matchmaker in ChimeraX[2] on BtBamG and BtBamG2 (Bt1785); Cα-Cα RMSD was 1.01 Å between 249 pruned atom pairs. **b**, Superposition of the BtBamH cryo-EM structure and the Bt1786 AF3 prediction. The superposition was generated using Matchmaker in ChimeraX on BtBamH and BtBamH2 (Bt1786); Cα-Cα RMSD was 1.25 Å between 67 pruned atom pairs, and 15.41 Å between all 385 pairs.

Supplementary Movie Legends

Supplementary Movie 1. 3D variability analysis results showing cryo-EM density variability along three modes. 20 volumes per mode were reconstructed and filtered to 4 Å.

Supplementary Movie 1. The movie shows a trajectory starting from the PgBAM conformation, morphing to the BtBAM conformation, and back to the PgBAM conformation. The trajectory was generated using the 'morph' command in ChimeraX. The protein models are displayed as coloured solvent-accessible surfaces.

Supplementary Tables

Supplementary Table 1. *B. theta bamA-his* and *bamA-hisΔbamH* pulldown proteomics results compared to the wild-type strain. Significant hits are coloured red (enriched relative to wild type) or blue (depleted relative to wild type).

Supplementary Table 2. Cryo-EM data collection, processing and model refinement parameters.

	BtBAM			PgBAM	
**Data collection**					
Electron microscope	Titan Krios			Titan Krios	
Voltage (kV)	300			300	
Spherical aberration (μm)	2.7			2.7	
Camera	Falcon 4i (counting)			Gatan K3	
Energy filter	Selectris (10 eV slit)			BioQuantum (20 eV slit)	
Magnification	165,000x			105,000x	
Pixel size (Å)	0.74			0.84	
Total dose ($e^-/Å^2$)	35			41	
Defocus range (μm)	-0.8 to -2.0			-0.6 to 1.5	
Number of movies collected	13,558			11,459	
**Image Processing**	**Class 1**	**Class 2**		**Class 1**	**Class 2**
Symmetry	C1	C1		C1	C1
Initial number of particles	2,266,553	2,266,553		4,830,281	4,830,281
Final number of particles	48,834	56,321		86,584	13,929
Global resolution (FSC = 0.143)	3.28 Å	3.46 Å		3.24	4.26
Map sharpening B-factor ($Å^2$)	-58.0	-64.9		-82.6	-49.3
**Refinement**	**BtBamHIJK**	**BtBamADG**	**Complete BtBAM**	**PgBAM**	**Class 2**
Model composition					
Non-hydrogen atoms	10,517	9,004	19,521	15,298	-
Protein residues	1,325	1,119	2,444	1,917	-
R.m.s. deviations					
Bonds lengths (Å)	0.004	0.005	0.006	0.005	-
Bond angles (˚)	0.792	0.964	0.934	0.785	-
Validation					
MolProbity score	1.48	1.96	1.99	2.05	-
Clash score	2.70	11.45	11.21	10.48	-
Rotamer outliers (%)	0	0	0	0	-
Ramachandran plot					
Favoured (%)	93.52	94.31	93.51	91.74	-
Outliers (%)	0.08	0	0.04	0.21	-
PDB	9HIS	9HIV	9HJ3	9HJM	-
EMDB	EMD-52200	EMD-52202	EMD-52209	EMD-52218	EMD-52219

Supplementary Table 3. BlastP search results using BamG sequence as query against the NCBI RefSeq database.

Supplementary Table 4. *B. theta bamH* deletion *vs* wild type total membrane fraction proteomics results shown as protein abundance differences. OMP categories are coloured as follows: yellow, SLP; green, β-barrel; blue, SusC (*i.e*. a barrel that assembles with one or more SLPs).

Supplementary Table 5. *P. gingivalis bamH* deletion *vs* wild type whole cell proteomics results shown as protein abundance differences. OMP categories are coloured as follows: yellow, SLP; green, β-barrel; blue, SusC (*i.e.* a barrel that assembles with one or more SLPs; grey, T9SS component; purple, T9SS substrate.

Supplementary Table 6. Strains used in this study.

Strain	Relevant genotype	Source
	E. coli	
**TOP10**	F- *mcrA* Δ(*mrr-hsd*RMS-*mcr*BC) Φ80*lac*ZΔM15 Δ*lac*X74 *rec*A1 *ara*D139 Δ(*araleu*)7697 *gal*U *gal*K *rps*L (StrR) *end*A1 *nup*G	Invitrogen
**S17-1λpir**	*TpR SmR recA thi pro hsdR-M+RP4:2-Tc:Mu:Km* Tn7 *λpir.*	ATCC
	P. gingivalis	
**ATCC33277**	Wild type	[3]
**RagB-8His ATCC33277**	*ragB* p.N^{503}_extHHHHHHHH (Tet^{R})	[4]
ΔbamH **ATCC33277**	*ΔbamH* (NCBI:PGN_1735)(Tet^{R})	This study
ΔbamI **ATCC33277**	*ΔbamI* (NCBI:PGN_0296)(Tet^{R})	This study
ΔbamK **ATCC33277**	*ΔbamK*(NCBI:PGN_1188)(Tet^{R})	This study
**BamH-7His ATCC33277**	*bamH* (NCBI:PGN_1735) p.N^{455}_extHHHHHHH (Tet^{R})	This study
**RagB-8His in *ΔbamH* ATCC33277**	*ΔbamH ragB* p.N^{503}_extHHHHHHHH (Em^{R})(Tet^{R})	This study
	B. theta	
**VPI-5482 *tdk^{-}***	*tdk^{-}*	[5]
**Bt1761$_{his}$**	*tdk^{-} bt1760$_{D42A}$ bt1761$_{his}$*	[6]
**Bt1761$_{his}$ *ΔbamH***	*tdk^{-} bt1760$_{D42A}$ bt1761$_{his}$ bt4306^{-}*	This study
**BtBamA$_{his}$**	*tdk^{-} bt3725$_{his}$*	This study
**BtBamA$_{his}$ *ΔbamH***	*tdk^{-} bt3725$_{his}$ bt4306^{-}*	This study
ΔbamI	*tdk^{-} bt3727^{-}*	This study
**Bt1927-ON**	*tdk^{-} bt1927-ON*	[7]
**Bt1927-ON *ΔbamH***	*tdk^{-} bt1927-ON bt4306^{-}*	This study
**SusA*$_{his}$***	*tdk^{-} bt3704$_{his}$*	J. Abellon-Ruiz (unpublished)

Supplementary Table 7. Plasmids used in this study.

Plasmids		
**Plasmid**	**Relevant features**	**Source**
**pUC19**	*E. coli* cloning vector, Amp^R	Thermo Fisher Scientific
**pExchange**	Plasmid for *Bacteroides* spp. genome engineering by allelic exchange.	[5]
ΔbamH	Plasmid for deletion of *P. gingivalis bamH* gene, derivative of pUC19	This study
ΔbamI	Plasmid for deletion of *P. gingivalis bamI* gene, derivative of pUC19	This study
ΔbamK	Plasmid for deletion of *P. gingivalis bamK* gene, derivative of pUC19	This study
**BamHall**	Master plasmid for *P. gingivalis bamH* modifications, derivative of pUC19	This study
**BamH-7His**	Plasmid for insertion of His_7-tag at C-terminal of *P. gingivalis* BamH, used for purification of the BAM complex, derivative of BamHall	This study
**pEx-*bamA*-his**	Plasmid for inserting a His7-tag at the N-terminus of *B. theta* BamA, derivative of pExchange	This study
**pEx-*bamH*-KO**	Plasmid for deletion of *B. theta bamH* gene, derivative of pExchange	This study
**pEx-*bamI*-KO**	Plasmid for deletion of *B. theta bamI* gene, derivative of pExchange	This study

Supplementary References

1. Abramson, J. *et al.* Accurate structure prediction of biomolecular interactions with AlphaFold 3. *Nature* **630**, 493–500 (2024).
2. Pettersen, E. F. *et al.* UCSF ChimeraX: Structure visualization for researchers, educators, and developers. *Protein Science* **30**, 70–82 (2021).
3. Naito, M. *et al.* Determination of the Genome Sequence of *Porphyromonas gingivalis* Strain ATCC 33277 and Genomic Comparison with Strain W83 Revealed Extensive Genome Rearrangements in *P. gingivalis*. *DNA Research* **15**, 215–225 (2008).
4. Madej, M. *et al.* Structural and functional insights into oligopeptide acquisition by the RagAB transporter from *Porphyromonas gingivalis*. *Nature Microbiology* **5**, 1016–1025 (2020).
5. Koropatkin, N. M., Martens, E. C., Gordon, J. I. & Smith, T. J. Starch Catabolism by a Prominent Human Gut Symbiont Is Directed by the Recognition of Amylose Helices. *Structure* **16**, 1105–1115 (2008).
6. White, J. B. R. *et al.* Outer membrane utilisomes mediate glycan uptake in gut Bacteroidetes. *Nature* **618**, 583–589 (2023).
7. Taketani, M., Donia, M. S., Jacobson, A. N., Lambris, J. D. & Fischbach, M. A. A phase-variable surface layer from the gut symbiont *Bacteroides thetaiotaomicron*. *mBio* **6**(5):e01339-15 (2015).
