# Supplementary material for: Structure of a distinct β-barrel assembly machinery complex in the Bacteroidota
Source: Nat Microbiol. 2025 Oct 1;10(11):2845–59. doi: 10.1038/s41564-025-02132-2 (PMC12578637; doi:10.1038/s41564-025-02132-2)
Supplement: Supplementary file 2 — Reporting Summary [file 41564_2025_2132_MOESM2_ESM.pdf]

nature portfolio

Corresponding author(s): Bert van den Berg

Last updated by author(s): Aug 13, 2025

Reporting Summary

Nature Portfolio wishes to improve the reproducibility of the work that we publish. This form provides structure for consistency and transparency in reporting. For further information on Nature Portfolio policies, see our Editorial Policies and the Editorial Policy Checklist.

Statistics

For all statistical analyses, confirm that the following items are present in the figure legend, table legend, main text, or Methods section.

n/a	Confirmed	
☐	☒	The exact sample size (n) for each experimental group/condition, given as a discrete number and unit of measurement
☐	☒	A statement on whether measurements were taken from distinct samples or whether the same sample was measured repeatedly
☐	☒	The statistical test(s) used AND whether they are one- or two-sided<br>*Only common tests should be described solely by name; describe more complex techniques in the Methods section.*
☒	☐	A description of all covariates tested
☒	☐	A description of any assumptions or corrections, such as tests of normality and adjustment for multiple comparisons
☐	☒	A full description of the statistical parameters including central tendency (e.g. means) or other basic estimates (e.g. regression coefficient) AND variation (e.g. standard deviation) or associated estimates of uncertainty (e.g. confidence intervals)
☐	☒	For null hypothesis testing, the test statistic (e.g. F, t, r) with confidence intervals, effect sizes, degrees of freedom and P value noted<br>*Give P values as exact values whenever suitable.*
☒	☐	For Bayesian analysis, information on the choice of priors and Markov chain Monte Carlo settings
☒	☐	For hierarchical and complex designs, identification of the appropriate level for tests and full reporting of outcomes
☒	☐	Estimates of effect sizes (e.g. Cohen's d, Pearson's r), indicating how they were calculated

Our web collection on statistics for biologists contains articles on many of the points above.

Software and code

Policy information about availability of computer code

Data collection	Gatan Microscopy Suite v3.4.3, Evosep One HyStar Driver 2.3.57.0, Compass HyStar v6.2, BLASTP 2.12.0+
Data analysis	cryoSPARC v4.5.3, Coot v0.9.8.8, Phenix v1.20.1-4487, AlphaFold v2, AlphaFold v3 (web server), UCSF ChimeraX v1.6-v1.9, ConSurf (web server), RStudio 2024.12.0+467, Microsoft Excel 365, DIA-NN v1.8.2.27, KNIME v5.1.2, FragPipe-Analyst, Limma, Enzyme Function Initiative - Enzyme Similarity Tool, SeaView v5.0.5, IQ-TREE.

For manuscripts utilizing custom algorithms or software that are central to the research but not yet described in published literature, software must be made available to editors and reviewers. We strongly encourage code deposition in a community repository (e.g. GitHub). See the Nature Portfolio guidelines for submitting code & software for further information.

Data

Policy information about availability of data

All manuscripts must include a data availability statement. This statement should provide the following information, where applicable:

- Accession codes, unique identifiers, or web links for publicly available datasets
- A description of any restrictions on data availability
- For clinical datasets or third party data, please ensure that the statement adheres to our policy

Pulldown proteomic mass spectrometry data sets and results files are referenced in ProteomeXchange (PXD058163) and available to download from MassIVE (MSV000096498) [doi:10.25345/C57W67J00]. Pre-publication access can be obtained with the following link ftp://MSV000096498@massive.ucsd.edu (username

MSV000096498, password S73xCTORvm9SM2m7). B. theta membrane and P. gingivalis whole cell mass spectrometry proteomics data have been deposited to the ProteomeXchange Consortium via the PRIDE partner repository with the dataset identifiers PXD058903 for B. theta and PXD058905 for P. ginigivalis. (reviewers can access the dataset by logging in to the PRIDE website using the following account details for B. theta: username: reviewer_pxd058903@ebi.ac.uk,password afLrMXZevbBq; or P. gingivalis: project accession: PXD058905, token: eAL4YgXN00lJ. Cryo-EM reconstructions and atomic coordinate files with the indicated accession numbers have been uploaded to the Electron Microscopy Data Bank (EMDB) and the Protein Data Bank (PDB): BtBAM class 1 (EMD-52200 and 9HIS), BtBAM class 2 (EMD-52202 and 9HIV), BtBAM composite map and model (EMD-52209 and 9HJ3), PgBAM class 1 (EMD-52218 and 9HJM), and PgBAM class 2 (EMD-52219).

Research involving human participants, their data, or biological material

Policy information about studies with human participants or human data. See also policy information about sex, gender (identity/presentation), and sexual orientation and race, ethnicity and racism.

Reporting on sex and gender	N/A
Reporting on race, ethnicity, or other socially relevant groupings	N/A
Population characteristics	N/A
Recruitment	N/A
Ethics oversight	N/A

Note that full information on the approval of the study protocol must also be provided in the manuscript.

Field-specific reporting

Please select the one below that is the best fit for your research. If you are not sure, read the appropriate sections before making your selection.

☒ Life sciences ☐ Behavioural & social sciences ☐ Ecological, evolutionary & environmental sciences

For a reference copy of the document with all sections, see nature.com/documents/nr-reporting-summary-flat.pdf

Life sciences study design

All studies must disclose on these points even when the disclosure is negative.

Sample size	No statistical methods were used to predetermine sample sizes. Sample sizes were set to 2 or 3 (or more) to assess and ensure standard reproducibility.<br>For cryo-EM, the collected datasets contained enough particles for structure determination.
Data exclusions	Cryo-EM movies with unacceptable CTF parameters, average intensity and relative ice thickness were excluded from data analysis. Particle images that did not yield good 2D class averages or 3D reconstructions were excluded from single particle analysis.
Replication	Structure determination experiments were not repeated, as is standard practice in the field.<br>Biochemical and growth assays were repeated at least three times on different days to ensure reproducibility.
Randomization	Particle stacks were randomly split into two independent sets during 3D volume reconstruction in single particle analysis.<br>For all other experiments, randomization was not possible.
Blinding	Knowing the identities of the samples was required for carrying out the analyses, and no blinding was performed as part of data analysis.<br>Investigators were not blinded to group allocation, as no grouping was needed for this study.

Reporting for specific materials, systems and methods

We require information from authors about some types of materials, experimental systems and methods used in many studies. Here, indicate whether each material, system or method listed is relevant to your study. If you are not sure if a list item applies to your research, read the appropriate section before selecting a response.

Materials & experimental systems

n/a	Involved in the study	
☐	☒	Antibodies
☒	☐	Eukaryotic cell lines
☒	☐	Palaeontology and archaeology
☒	☐	Animals and other organisms
☒	☐	Clinical data
☒	☐	Dual use research of concern
☒	☐	Plants

Methods

n/a	Involved in the study	
☒	☐	ChIP-seq
☒	☐	Flow cytometry
☒	☐	MRI-based neuroimaging

Antibodies

Antibodies used

anti-HmuY polyclonal antibodies (concentration used- 1 µg/ml) - provided by Prof. Teresa Olczak (University of Wroclaw, Poland), HRP-conjugated anti-rabbit antibodies (Sigma Aldrich, A0545) - dilution 1:20,000

Validation

anti-HmuY polyclonal antibodies were valideted in published manuscript: doi: 10.1371/journal.pone.0117508. eCollection 2015 HRP-conjugated anti-rabbit antibodies (Sigma Aldrich, A0545) were used according to manufacturer's recommendations.

Plants

Seed stocks

Report on the source of all seed stocks or other plant material used. If applicable, state the seed stock centre and catalogue number. If plant specimens were collected from the field, describe the collection location, date and sampling procedures.

Novel plant genotypes

Describe the methods by which all novel plant genotypes were produced. This includes those generated by transgenic approaches, gene editing, chemical/radiation-based mutagenesis and hybridization. For transgenic lines, describe the transformation method, the number of independent lines analyzed and the generation upon which experiments were performed. For gene-edited lines, describe the editor used, the endogenous sequence targeted for editing, the targeting guide RNA sequence (if applicable) and how the editor was applied.

Authentication

Describe any authentication procedures for each seed stock used or novel genotype generated. Describe any experiments used to assess the effect of a mutation and, where applicable, how potential secondary effects (e.g. second site T-DNA insertions, mosiacism, off-target gene editing) were examined.
