# Supplementary figures and images for: Structure of a distinct β-barrel assembly machinery complex in the Bacteroidota
Source: Nat Microbiol. 2025 Oct 1;10(11):2845–59. doi: 10.1038/s41564-025-02132-2 (PMC12578637; doi:10.1038/s41564-025-02132-2)

M *bamA*-His

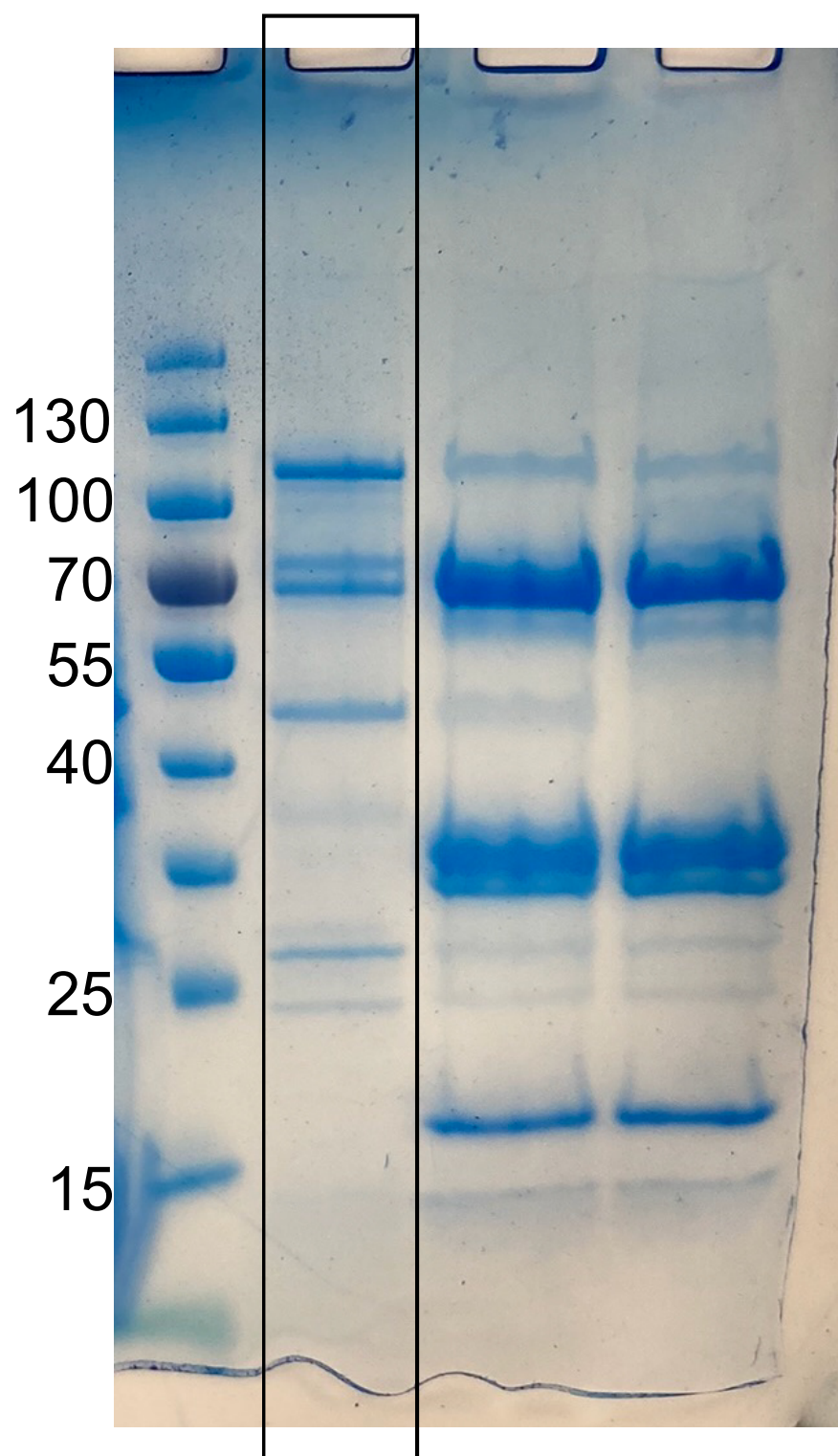

Supplement: Supplementary file 7 — Uncropped gel. [file 41564_2025_2132_MOESM7_ESM.pdf]

*bamA*-His  
 $\Delta$ *bamG*

*bamA*-His

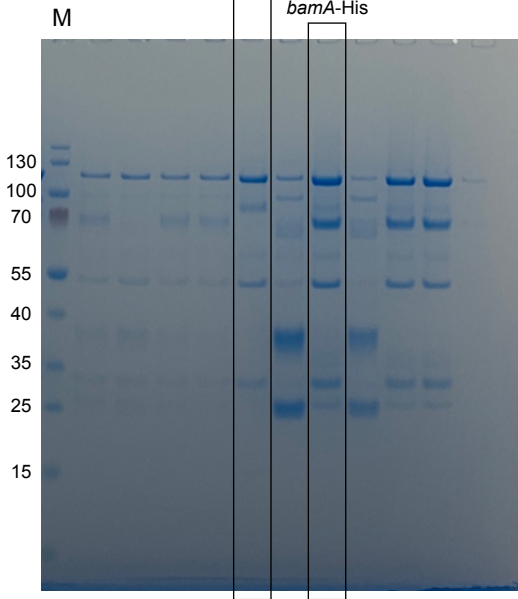

Supplement: Supplementary file 8 — Uncropped gel. [file 41564_2025_2132_MOESM8_ESM.pdf]

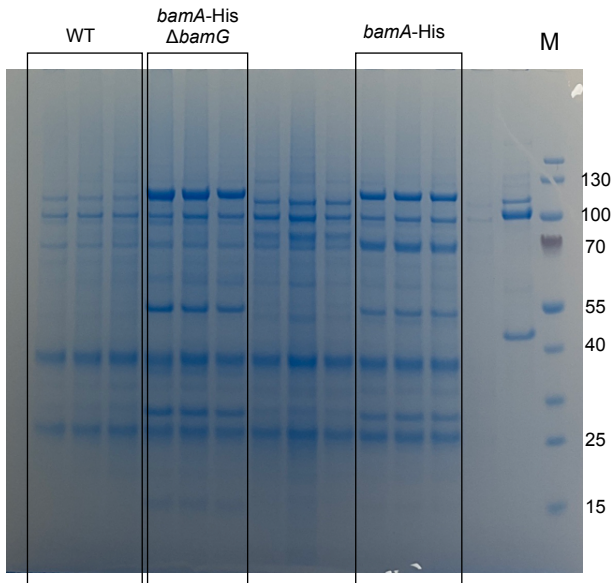

Supplement: Supplementary file 10 — Uncropped gel. [file 41564_2025_2132_MOESM10_ESM.pdf]
