# Supplementary material for: Structure of a distinct β-barrel assembly machinery complex in the Bacteroidota
Source: Nat Microbiol. 2025 Oct 1;10(11):2845–59. doi: 10.1038/s41564-025-02132-2 (PMC12578637; doi:10.1038/s41564-025-02132-2)

Figure 5b uncropped blots

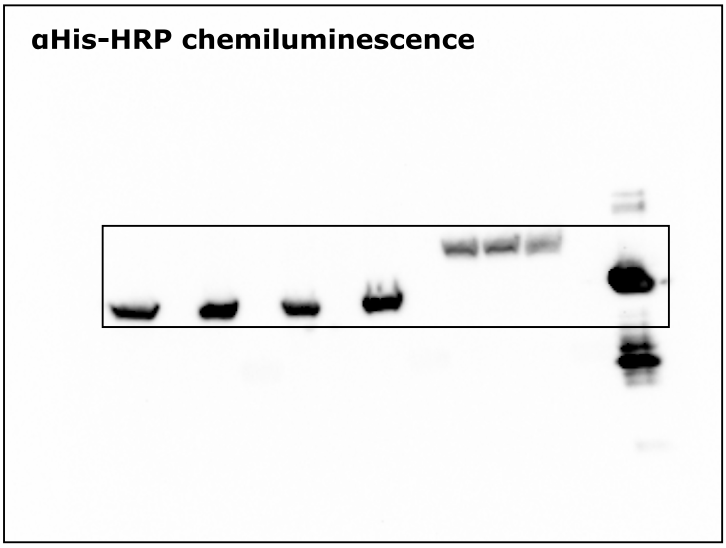

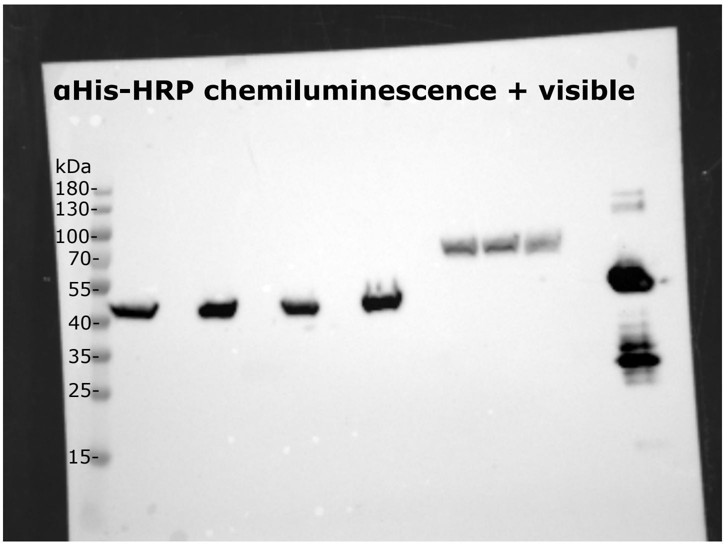

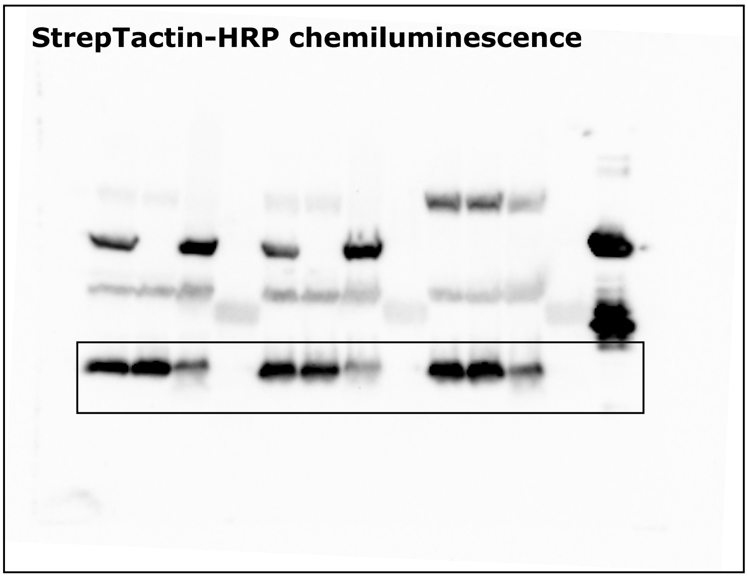

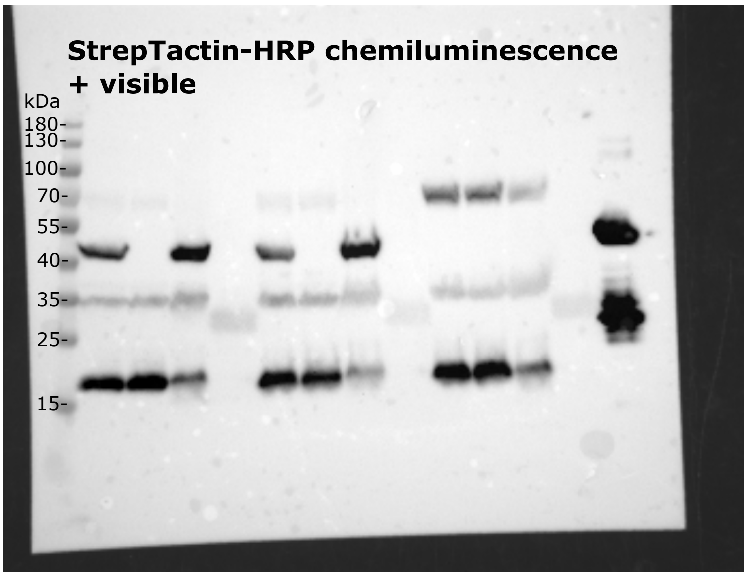

Figure 5e uncropped blots

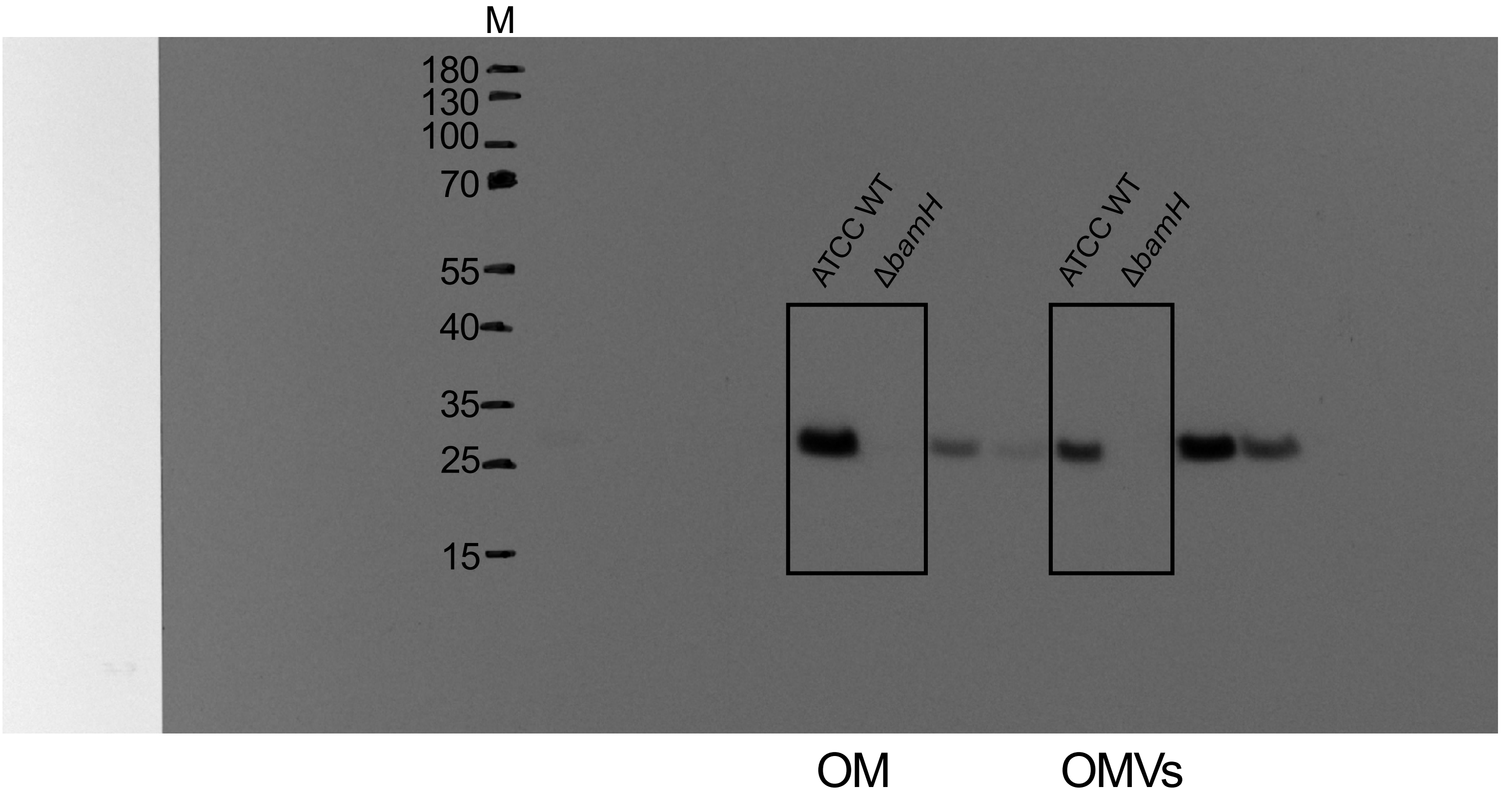

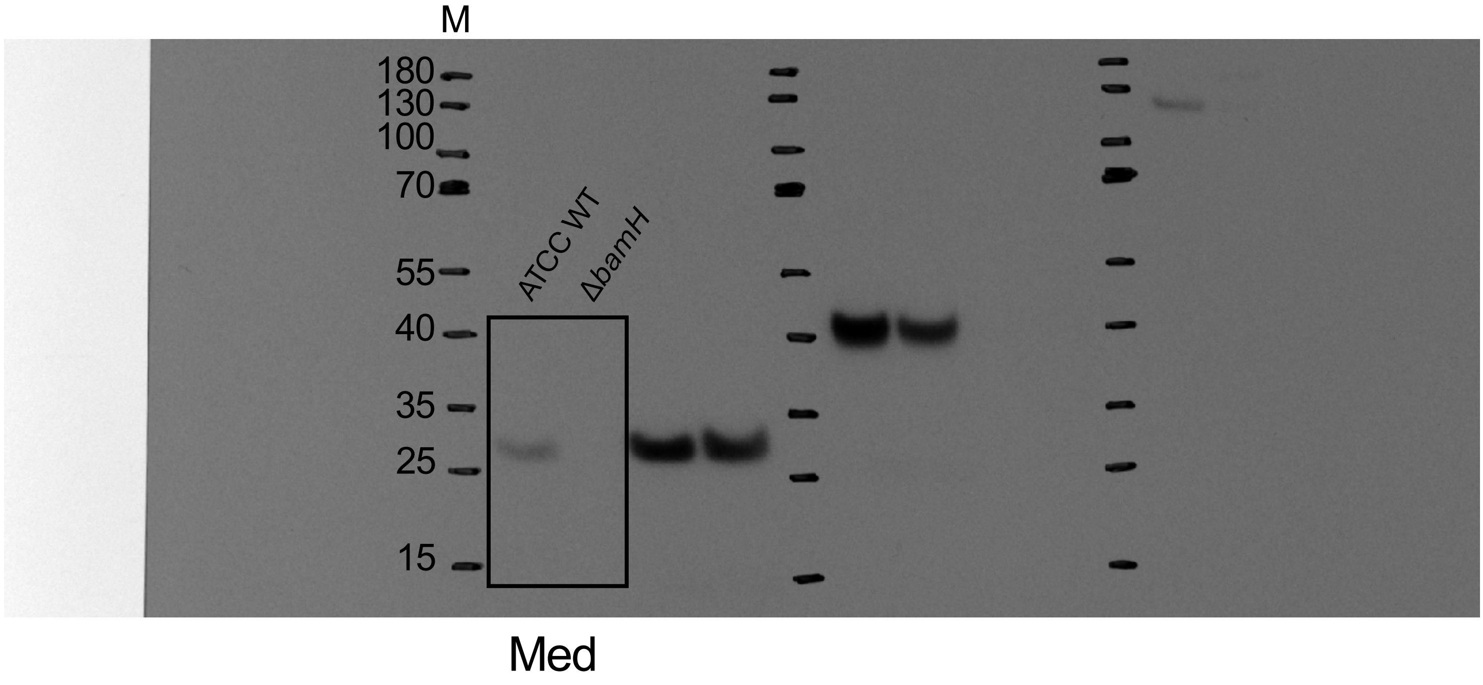

Figure 5e Uncropped gel

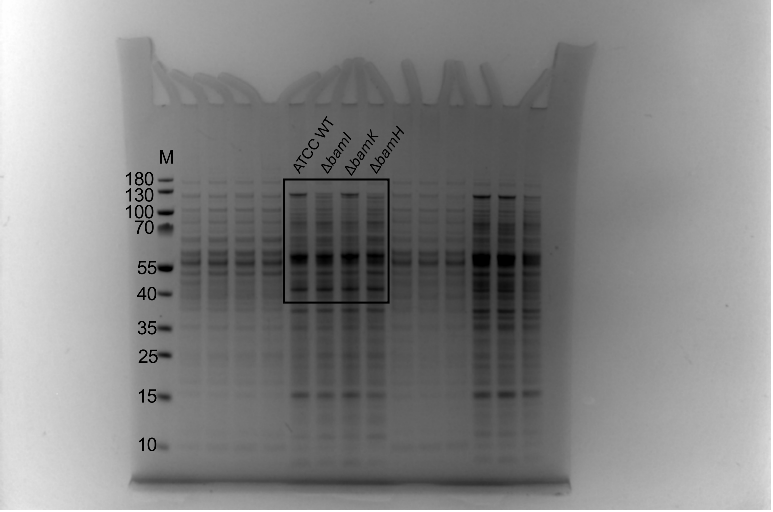

Figure 5h Uncropped gel

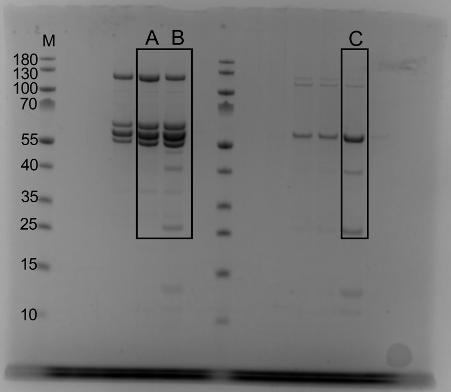

Supplement: Supplementary file 9 — Uncropped blots and gels. [file 41564_2025_2132_MOESM9_ESM.pdf]
